# Shortening the drug discovery pipeline: small molecule high content screening for lead discovery in neglected disease

**DOI:** 10.1186/1753-6561-5-S1-P38

**Published:** 2011-01-10

**Authors:** Jonathan Cechetto, Hee Kyoung Jeon, Jiyeon Jang, Doyoon Kwon, Thierry Christophe, Priscille Brodin, Gyongseon Yang, Jair Neto, Lucio Freitas Junior

**Affiliations:** 1 Screening Technology Platforms, Institut Pasteur Korea, Seongnam-si, 463-400, Korea; 2 Biology of Intracellular Pathogens, Institut Pasteur Korea, Seongnam-si, 463-400, Korea; 3 Center for Neglected Diseases, Institut Pasteur Korea, Seongnam-si, 463-400, Korea

## 

There is a pressing, global need for new therapies for neglected diseases such as Tuberculosis, Leishmaniasis and Chagas. Traditional lead discovery approaches, while effective have not been widely applied to neglected diseases as they are expensive and time consuming. At Institut Pasteur Korea, we have developed a core technology that enables the high content screening of hundreds of thousands of small molecules against the disease of interest. In doing so, we utilize relevant disease models that allow us to interrogate the disease in the context of the cell and the host-pathogen factors required for invasion, replication, persistence and release. The application of this core technology against Tuberculosis, Leishmaniasis and Chagas disease has resulted in the identification of novel, active compounds in less time and at less cost than traditional drug discovery methods. As these active compounds were identified in a cellular context, they can provide a more relevant starting point for new therapies.

